# Unsuccessful Tonsil and Adenoid Operations

**Published:** 1923-03

**Authors:** A. J. M. Wright

**Affiliations:** Surgeon to the Throat and Nose Department, Bristol General Hospital; Lecturer in Laryngology, Rhinology and Otology, University of Bristol


### UNSUCCESSFUL TONSIL AND ADENOID OPERATIONS.

BY

A. J. M. WRIGHT, M.B., F.R.C.S.,

*Surgeon to the Throat and Nose Department, Bristol General Hospital; Lecturer in Laryngology, Rhinology and Otology, University of Bristol.*

---

ALL operations are not successful, and it is a useful experience to try to assess the reason by studying our own and other people's failures. With this object in view, I have looked through the records of a group of cases of individuals who have consulted me with symptoms which had not been relieved by a previous operation, either by myself or another practitioner, for the removal of tonsils, adenoids, or both. I find that these cases naturally fall into two groups. Firstly, those in which the symptoms are due to the fact that the operation was imperfectly performed. I find that this was not a large group, only representing about a fifth of the total cases. Secondly, those in which the symptoms are due to some other condition, which is either a secondary result of the pre-existing tonsils and adenoids or is unconnected therewith.

I do not propose to deal further with the first group, except to say that the remedy obviously lies in the direction of greater care and thoroughness in carrying out the operation.

In the second group, of the conditions which are probably a direct result of tonsils and adenoids, nasal obstruction due to deflection of the septum, enlargement of the turbinals, or the two conditions combined, are those most commonly met with. We are all probably familiar with this type of patient, who when seen in early adult life presents a narrow nose with some degree of septal deviation, enlarged turbinals, high palate, crowded teeth, chronic mouth-breathing and frequently a deafness resulting from previous attacks of inflammation in the middle-ear. These conditions most frequently result in cases in which mouth-breathing has been practised for a considerable period, and their avoidance lies in the direction of earlier operation. I find that in this class of case the average age at which the operation was performed was 15 years. To get the best results an operation for tonsil and adenoids should be carried out as soon as decided symptoms are present. In the majority of cases the skeletal changes in the chronic mouth-breather take place at, or about, the second dentition, so that it is important, where symptoms are present, to operate before this age.

After a period of prolonged mouth-breathing, where these deformities have taken place, while operation on the septum and turbinals may give some relief, the individual is frequently condemned to be a permanent mouth-breather, partly owing to the inefficient airway and partly to the confirmed habit.

Another group of cases is that in which nasal discharge persists after an efficient operation. Such discharge in a child may be either the result of a suppurative rhinitis or of infection of the nasal sinuses, associated with adenoids as the primary condition. The removal of adenoids will usually clear up such a secondary rhinitis or sinus suppuration. A residue of cases is left, however, in which the rhinitis or sinus infection persists in spite of removal of the adenoids. Where sinus suppuration persists an intranasal operation will usually clear it up. On the other hand, some cases of suppurative rhinitis are only kept under control by nasal medication, and eventually after puberty are recognised as cases of typical atrophic rhinitis or ozæna. In cases in which a watery discharge with attacks of sneezing persists, often associated with asthma, the condition will usually be found to be one of so-called vasomotor rhinitis. This is an affection of the nasal mucous membrane, similar to that of the bronchial mucosa in asthma. These cases are, as a rule, not improved by the removal of a small amount of adenoids.

Inflammatory infections of the ears probably constitute the most important indication for the removal of tonsils and adenoids. When the operation is done sufficiently early the results are good. As is to be expected, however, a proportion of cases return for treatment to the ears, the damage which has taken place before operation being too extensive to permit of a complete recovery. There is, however, one other class of case in which it will be found that the deafness is due to some other condition and is not the result of tonsils and adenoids, as, for example, a nerve deafness or otosclerosis. I have seen several cases in which great disappointment was occasioned by the lack of any improvement in the ear condition after operation which might have been avoided by a more careful aural examination.

We are all familiar with the fact that children with adenoids tend to have a typical intonation of the voice. On the other hand, it is a very frequent mistake to regard adenoids as the cause of gross speech defects. In one type of such defect, which is not very uncommon, the voice resembles that of an individual with a cleft palate, and this is probably at any rate partly due to a defect in the development of the palate, although no actual cleft exists. While I agree that it is very important to ensure that any child presenting a speech defect has its airway clear, it is of equal importance, if it is thought advisable to operate on such a case, to ensure that the relatives clearly understand that the operation cannot of itself cure the condition. I believe that not infrequently an attempt is made to remove non-existent adenoids in such cases. Cases in which there is a greater or less degree of mental deficiency require somewhat similar handling. A mentally backward child naturally requires every assistance that we can give it, but it is not a very uncommon mistake to regard the vacant expression and open mouth of imbecility as the results of adenoids. Two cases of this class which particularly impressed me have been children which, I believe, were suffering more from a mild degree of cretinism than from adenoids.

Chronic bronchitis in a child is not infrequently secondary to the infection of adenoids, and this fact is becoming increasingly recognised. On the other hand, I have recently seen a case in which the wheezing of a chronic bronchitis, probably associated with some degree of asthma, led to a mistaken diagnosis of adenoids.

To sum up. A consideration of cases in which symptoms are not relieved by operation for tonsils and adenoids shows that this is only occasionally due to the inefficiency of the operation. In a larger proportion of cases it is due to insufficient care in the previous diagnosis, and more attention should be paid to the previous examination and weighing of symptoms. The operation is so frequently done, that it is tending to become rather a matter of routine.

